# IgG subclass and Fc glycosylation shifts are linked to the transition from pre- to inflammatory autoimmune conditions

**DOI:** 10.3389/fimmu.2022.1006939

**Published:** 2022-11-03

**Authors:** Jana Sophia Buhre, Mareike Becker, Marc Ehlers

**Affiliations:** ^1^ Laboratories of Immunology and Antibody Glycan Analysis, Institute of Nutritional Medicine, University of Lübeck and University Hospital Schleswig-Holstein, Lübeck, Germany; ^2^ Department of Dermatology, Allergology, and Venereology, University of Lübeck and University Hospital Schleswig-Holstein, Lübeck, Germany; ^3^ Airway Research Center North, German Center for Lung Research (DZL), University of Lübeck, Lübeck, Germany

**Keywords:** autoimmunity, IgG subclass, IgG glycosylation, pre-autoimmune disease stage, inflammatory autoimmune disease stage

## Abstract

A crucial factor for the development of inflammatory autoimmune diseases is the occurrence of antibodies directed against self-tissues and structures, which leads to damage and inflammation. While little is known about the cause of the development of mis-directed, disease-specific T and B cells and resulting IgG autoantibody responses, there is increasing evidence that their induction can occur years before disease symptoms appear. However, a certain proportion of healthy individuals express specific IgG autoantibodies without disease symptoms and not all subjects who generate autoantibodies may develop disease symptoms. Thus, the development of inflammatory autoimmune diseases seems to involve two steps. Increasing evidence suggests that harmless self-directed T and B cell and resulting IgG autoantibody responses in the pre-autoimmune disease stage might switch to more inflammatory T and B cell and IgG autoantibody responses that trigger the inflammatory autoimmune disease stage. Here, we summarize findings on the transition from the pre-disease to the disease stage and vice versa, e.g. by pregnancy and treatment, with a focus on low-/anti-inflammatory versus pro-inflammatory IgG autoantibody responses, including IgG subclass and Fc glycosylation features. Characterization of biomarkers that identify the transition from the pre-disease to the disease stage might facilitate recognition of the ideal time point of treatment initiation and the development of therapeutic strategies for re-directing inflammatory autoimmune conditions.

## Introduction

Inflammatory autoimmune diseases are a worldwide threat to health and show an increasing prevalence (1). Although tumor-reactive IgG autoantibodies (autoAbs) can mediate beneficial roles in eliminating tumor cells, IgG autoantibodies are often key players in the induction of inflammatory autoimmune diseases. Accordingly, depletion of B cells with rituximab (monoclonal anti-CD20 Ab) often improves inflammatory autoimmune disease conditions (2). Interestingly, autoimmune patients can start to express IgG autoAbs years before developing specific clinical symptoms (3–5). Furthermore, a certain proportion of healthy individuals express specific IgG autoAbs without disease symptoms (6, 7).

**Figure 1:**
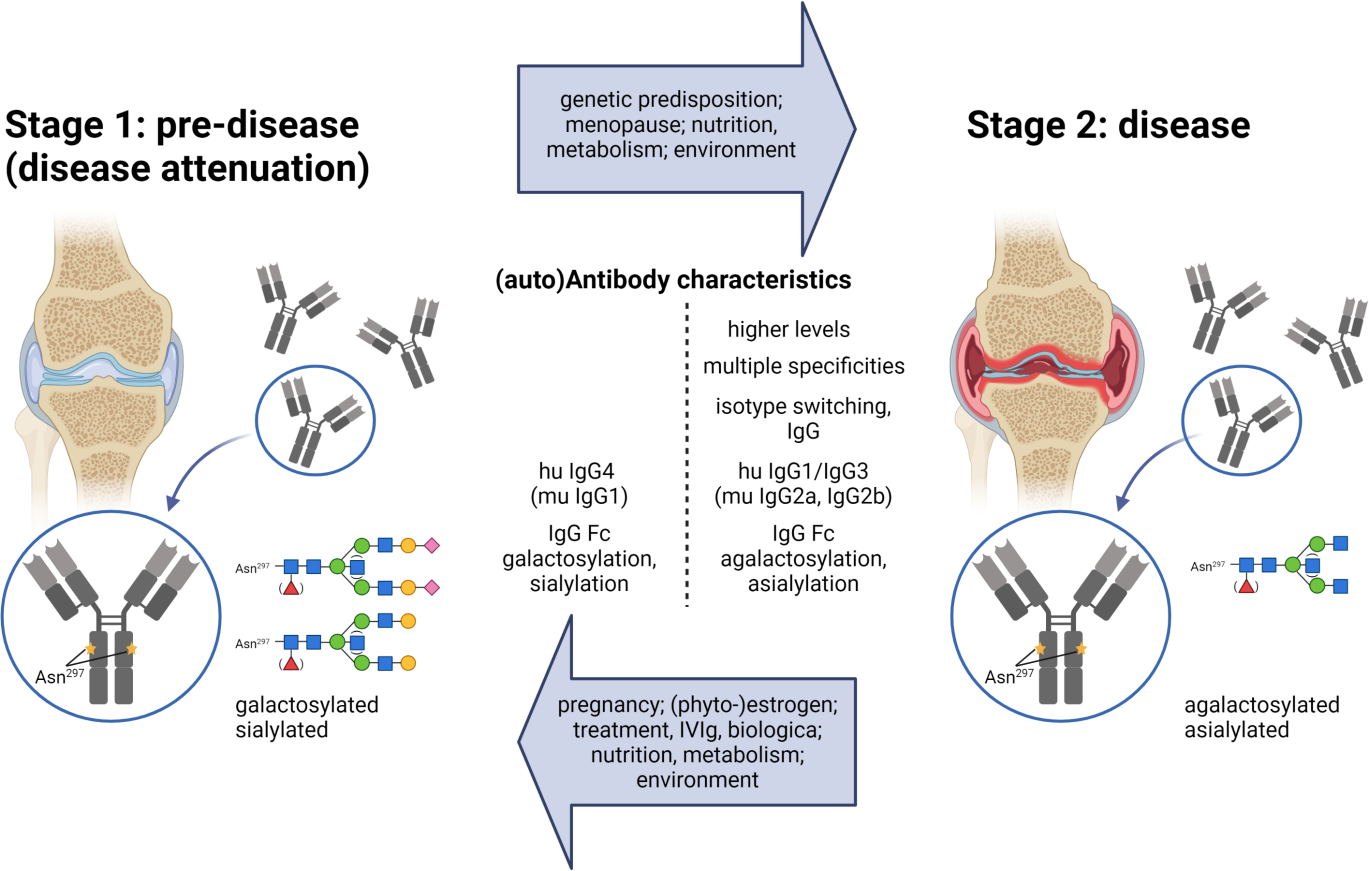
The two-stage model for the development of inflammatory autoimmune diseases. In Stage 1, the pre-disease state, autoAbs are present, but there are no disease symptoms. Due to reasons indicated in the upper arrow, the condition shifts to an inflammatory stage 2, where disease symptoms occur. Enhanced antibody titers, multiple specificities, IgG class switching, and shifts in IgG subclasses and Fc glycosylation patterns are described for autoantibodies in the disease state (described in the text). Factors shown in the lower arrow are confirmed, suggested, or discussed to redirect an inflammatory antibody response into the direction of the low-/non-inflammatory pre-disease state. IgG Fc glycosylation patterns: blue, *N*-acetylglucosamine (GlcNAcs); green, mannose; red, fucose, yellow, galactose; purple, sialic acid.

The occurrence of IgG autoAbs at an early pre-disease stage was initially described for (seropositive) rheumatoid arthritis (RA) (**Figure 1**). Anti-citrullinated peptide IgG autoAbs can be detected years before RA disease symptoms develop (3).

Another example is diabetes mellitus type 1. In an interesting study, several thousand healthy infants without a first-degree family history of diabetes were screened for autoAbs typical of diabetes type-1 (4). A total of 155 of 7787 infants were repeatedly screened positive for such autoAbs. Several years later, 26 of these 155 autoAb-positive infants and only two of the autoAb-negative infants had developed diabetes type 1 (4).

These studies lead to two very important conclusions. First, individuals generating specific IgG autoAbs also have a higher risk of developing the specific inflammatory autoimmune disease (3, 4). This finding opens the possibility of identifying pre-disposed individuals before developing disease symptoms by close-meshed monitoring (5). Second, not all individuals with specific IgG autoAbs develop the respective inflammatory autoimmune disease, at least not at a short interval.

Accordingly, it is increasingly assumed that the development of inflammatory autoimmune diseases has to undergo two steps. In step one, and for incompletely understood reasons, tolerance mechanisms fail, and an autoantigen-specific T and B cell response leads to detectable IgG autoAbs. Several findings suggest that these IgG autoAbs, however, might be harmless in the pre-autoimmune disease stage and do not induce any clinical signs. In the second step, this specific immune response might shift in some but not all individuals to a more inflammatory T and B (T/B) cell and IgG Ab response that gives rise to various disease conditions (inflammatory autoimmune disease stage) often years after step one (**Figure 1**) (3, 4, 8–14). Such a shift might likely be dependent on genetic predispositions and environmental factors determining inflammatory conditions. Steps one and two might also occur simultaneously.

Here, we summarize findings on the transition from the pre-disease to the disease stage and vice versa, e.g. by pregnancy and treatment, with a focus on low-/anti-inflammatory versus pro-inflammatory IgG autoAb responses.

## Low versus high inflammatory IgG (auto) antibody responses

The inflammatory severity of IgG (auto)Abs may be dependent on the IgG autoAb-specific subclass and glycosylation pattern as well as the total IgG glycosylation pattern. Knowledge about these determinants will be described and discussed in the following paragraphs.

### Activating versus inhibitory IgG subclasses

The following functional IgG subclass pairs between human and mouse have been identified: human (hu) IgG1 and murine (mu) IgG2a (IgG2c); hu IgG2 and mu IgG3; hu IgG3 and mu IgG2b; and hu IgG4 and mu IgG1 (15).

Human IgG1 and IgG3 as well as murine IgG2a and IgG2b show the highest affinities to the classical activating FcyRs and C1q, the starting molecule of the classical complement activation pathway (16–18). These IgG subclasses seem to be able to form hexamers facilitating the interaction with the six-arm C1q molecule (15, 19–23). Human IgG2 and murine IgG3 hardly interact with classical FcyRs and C1q and their effector functions are mostly unclear (16–18). They are induced for instance by T cell-independent antigens. Furthermore, recent studies have shown that murine IgG3 can form complexes that induce nephritis (24).

Human IgG4 and murine IgG1 show higher affinities to the only classical IgG inhibitory receptor FcyRIIB than to their classical activating FcyRs (16–18). Furthermore, human IgG4 and murine IgG1 cannot activate C1q, but seem to be able to disturb hexamer formation of the C1q-activating IgG subclasses (15). Murine IgG1 also inhibits the formation of murine IgG3 complexes (24). Furthermore, human IgG4 and murine IgG1 can generate Fab arm exchange meaning that heavy chains with different specificities can dimerize, which reduces their ability to form immune complexes (25).

Thus, human IgG1/IgG3 and murine IgG2a/IgG2b are the IgG subclasses with the highest potential to activate the immune system, whereas human IgG4 and murine IgG1 have less activating potential and can even inhibit the effector functions of human IgG1/IgG3 and murine IgG2a/IgG2b. Accordingly, inflammatory autoimmune diseases are often characterized by the appearance of the activating human IgG1/IgG3 and murine IgG2a/IgG2b subclasses, and the first studies showed that the autoantigen-specific IgG subclass shift from inhibitory to activating IgG subclasses is associated with higher inflammatory autoimmune conditions and vice versa that enrichment of the inhibitory human IgG4/murine IgG1 subclass can counteract inflammatory (auto)immune conditions (**Figure 1**) (10, 15, 24, 26). However, corresponding human studies are scarce and are needed to verify these observations. Other autoantigen-specific IgD, IgM, IgA and IgE isotypes might also be involved, an area that is less investigated and not the subject of this review. In addition, increased autoAb titers (14) and autoantigen specificities (4) might facilitate the transition from the pre- to the disease stage.

### Pro-inflammatory versus lower-/anti-inflammatory IgG Fc *N*-glycosylation patterns

The effector functions of IgG molecules are additionally linked to their type of Fc *N*-glycosylation attached to asparagine 297 (Asn297, *N*) of both heavy chains in the IgG Ab Fc region (27). A highly conserved biantennary glycan core structure consisting of *N*-acetylglucosamines (GlcNAcs) and mannoses can be further modified with a fucose, a bisecting GlcNAc and one or two galactose residues, each of which can be further capped by a sialic acid (27, 28) (**Figure 1**).

Autoantigen-specific agalactosylated (non-galactosylated and non-sialylated; G0) IgG Abs are linked to pro-inflammatory conditions in inflammatory (auto-) immune diseases, whereas attachment of galactose and sialic acid is related to fewer inflammatory or even anti-inflammatory conditions (**Figure 1**) (3, 8, 11–13, 28–39). In most studies, IgG Fc bisection also correlates with pro-inflammatory conditions (28). IgG Fc afucosylation in particular has been connected to a high tumor fighting potential (16, 40), but shows different trends in distinct inflammatory autoimmune diseases (28, 41–43).

Mechanistically, afucosylated IgG Abs show an increased affinity to certain classical activating FcyRs (40). The effector function of IgG Fc bisection has rarely been investigated (28). Sialylated IgG Abs have shown a decreased affinity to classical (murine) activating FcyRs (27). The described functions of galactose are controversial as galactosylated IgG Abs show an enhanced interaction with C1q (44), whereas IgG agalactosylation increases the induction of the lectin and alternative complement pathways (32, 45–47). However, the *in vivo* functions of differently glycosylated IgG Abs seem to be much more complex because single terminal glycan residues can also interact with glycan binding receptors, e.g., of the galectin, siglec and C-type lectin receptor families (34–36, 48–50). *In vivo*, immune inhibitory functions have been described for sialylated as well as terminal galactosylated antigen-specific and total IgG Abs (11, 27, 34–36, 38, 48–52). However, further studies are needed to solve the *in vivo* functions of differently glycosylated (auto)antigen-specific IgG (subclass) Abs.

Several studies have suggested that the transition from the pre-autoimmune disease stage to the inflammatory autoimmune disease stage is linked to decreasing IgG autoAb galactosylation and sialylation levels (3, 8, 9, 11–14). Interestingly, an increase in anti-citrullinated peptide IgG Fab glycosylation sites - very likely generated by somatic hypermutations - has recently been linked in addition to the shift from the pre-disease to the disease stage in the case of RA (53–55).

A reduction in autoantigen-specific human IgG4 and murine IgG1 galactosylation and sialylation levels seems to increase their inflammatory potential and might be an explanation for the appearance of autoantigen-specific IgG4-mediated inflammatory autoimmune diseases (15, 34, 36, 49, 52, 56–59).

#### Total IgG Fc *N*-glycosylation

In addition to autoantigen-specific serum IgG Fc glycosylation patterns, the corresponding total serum IgG Fc glycosylation patterns have been linked to inflammatory conditions, for instance in patients with RA. Autoantigen-specific IgG Abs not only enrich the total IgG, but rather the whole T and B cell responses seem to shift to a more inflammatory stage. Thus, low total serum IgG Fc galactosylation and sialylation levels correlate with severe inflammatory disease conditions in RA. In 2006, it was found in mouse studies that the therapeutic effect of IVIg (intravenous immunoglobulin; high amounts (2 g/kg/course) of pooled serum IgG from healthy donors to treat inflammatory diseases) is based on the sialylated total IgG subfraction (27, 28, 48, 60). Respectively, IVIg treatment may re-establish a lower inflammatory immune status.

The total IgG Fc glycosylation status seems to act as a huge immunological buffer system. Higher total IgG Fc sialylation levels seem to up-regulate classical inhibitory and down-regulate classical activating FcyRs (48, 52, 61), and further immune receptors might be affected. Accordingly, the total IgG Fc agalactosylation level is assumed to indicate the inflammatory status of each individual and increases with chronological and, in particular, biological age (62–65). The glycosylation pattern of total IgM and IgA Abs very likely also influences the immune status.

A change in total IgG Fc glycosylation during the transition from the pre-disease to the disease stage is controversial. A recent study described that low total IgG Fc galactosylation levels could also occur very early in the pre-disease stage of RA patients (66). However, total IgG Fc agalactosylation levels seem to be a risk factor for the development of RA.

#### Induction of IgG antibodies with low galactosylation and sialylation levels

Recent immunization studies have shown that different co-stimuli/adjuvants/inflammatory conditions induce distinct germinal center (GC) T and B cell responses that determine different expression levels of α2,6-sialyltransferase (St6gal1; the enzyme that adds sialic acid to IgG Fc parts) in GC-derived plasma cells (PCs) and corresponding IgG Fc sialylation levels (67). It is assumed that beta1,4-galactosyltransferase (B4galt1; the enzyme that adds galactose to IgG Fc parts) expression and corresponding IgG Fc galactosylation are regulated similarly in parallel (67). Accordingly, (auto)antigen-specific IgG Fc galactosylation and sialylation levels reflect the inflammatory immune status and can be used as biomarkers of the inflammatory potential of the running (auto)antigen-specific T and B cell response (67).

In this context and in the context of RA models, it has been shown that, in particular, IL-6, IL-27R-induced IFNγ-producing T_FH1_ and T_FH17_ cells contribute to the induction of low St6gal1 expression in GC B cells and corresponding PCs as well as low IgG Fc sialylation levels (12, 67). Abrogation of these signals has led to higher St6gal1 expression and higher IgG Fc galactosylation and sialylation levels (12, 67).

## Reasons for the switch from pre- to inflammatory conditions

In step one, tolerance mechanisms fail, and an autoantigen-specific T and B cell response leads to detectable IgG autoAbs. This step is even less understood than step two. A certain portion of individuals expressing specific IgG autoAbs might never develop specific disease symptoms. Others can switch from the pre- to the inflammatory stage (**Figure 1**). The reasons are still unclear and might occur individually. Increasing evidence suggests that the T/B cell and IgG autoAb responses induced in step one do not have to be pathogenic, but can switch to inflammatory, pathogenic IgG autoAb responses initiating the disease stage (3, 4, 8–14).

Three scenarios seem to be most likely for the transition. First, unfavorable genetic predispositions such as certain MHC alleles might favor such a switch (14, 68). Nevertheless, there is only a small overlap of the disease appearance between monozygotic twins (69), suggesting that additional factors might play an important role. Second, a specific event such as a severe lung infection/inflammation might switch the inflammatory status of the whole immune system for some days, which may lead to the alteration of a harmless autoantigen-specific T/B cell response in the pre-stage to an inflammatory T/B cell response. Third, the switch might occur slowly over time. Aging and increasing BMIs, for instance, shift the whole immune status as well as the total IgG Fc glycosylation level to a more inflammatory condition (62–65). Furthermore, unfavorable nutrition and a shift in the microbiome induced, for instance, by nutrition or antibiotics can influence the immune status (see also below). The accumulation of certain types of gut bacteria might then favor more inflammatory immune responses, e.g., by supporting the Th17 axis (70, 71). Slow shifts to more inflammatory immune conditions might also shift the autoantigen-specific T and B cell response to a more inflammatory state and induce the development of specific autoimmune disease symptoms.

## Pregnancy and estrogen lead to a return to less inflammatory (auto)immune conditions

To understand the shift from the pre- to the inflammatory stage, it might be helpful to analyze conditions when the inflammatory autoimmune stage returns to a less inflammatory stage in the direction of the pre-stage.

The most prominent case is likely pregnancy. Women with RA show less disease symptoms during pregnancy, a tolerogenic status established to inhibit immune attacks against the fetus (72, 73). During pregnancy, total as well as autoantigen-specific IgG Abs shift to higher Fc galactosylation and sialylation levels (72, 73). Notably, Fab glycosylation does not change during pregnancy (74). Understanding the changes in T/B cell and Ab responses during pregnancy in patients with inflammatory autoimmune diseases, such as RA, will facilitate understanding and recognition of the switch from the pre- disease to the disease stage.

Appropriately, the level of the sex hormone estrogen, which is highly upregulated during pregnancy and downregulated during menopause, positively correlates with IgG Fc galactosylation and sialylation levels in males and females (75). Furthermore, application of estrogen or phytoestrogens reduced inflammatory conditions, up-regulated B4galt1 and St6gal1 expression and enhances IgG Fc galactosylation and sialylation levels (75–77). In addition, phytoestrogens have been described to exert anti-inflammatory effects (78, 79).

## Early diagnosis

Healthy individuals with identified specific IgG autoAbs could be closely monitored to recognize any starting transition from the pre- to the inflammatory (auto)immune stage for starting therapies before clinical disease symptoms evolve. Therapies might then redirect the inflammatory autoantigen-specific T and B cell and Ab response back into the direction of the low-/non-inflammatory pre-stage (80). Total as well as autoantigen-specific IgG Fc galactosylation and sialylation levels seem to be promising biomarkers for characterizing any transition.

## Discussion of existing and potential new therapies

There are increasing therapeutic tools that reduce inflammatory (auto)immune conditions. Some, e.g. rituximab, deplete central immune cells such as B cells, and others, such as IVIg or monoclonal anti-TNFα and anti-IL-6 Abs, redirect pro-inflammatory to less inflammatory conditions. Alhough different therapeutics are available and frequently used, their anti-inflammatory mechanisms are often not completely understood. In the following section, we will address the anti-inflammatory potential of some existing therapeutics and discuss further possibilities to redirect inflammatory immune conditions or to maintain the pre-disease stage.

### IVIg

Different immune modulatory effects of IVIg have been described, one of which may be the re-establishment of the total IgG Fc glycosylation buffer system by increasing the proportion of the sialylated IgG Ab subfraction (27, 48, 81, 82). If a patient shows no response to IVIg therapy, higher amounts of IVIg might be necessary to re-establish a healthy/tolerogenic total IgG Fc galactosylation and sialylation level. In the meanwhile, there have been attempts to further modulate IVIg enzymatically by adding the maximal number of sialic acids (four) to one IgG molecule to enhance the anti-inflammatory properties and make the efficacy more consistent (83). However, when IVIg therapy is discussed, it must be mentioned that several anti-inflammatory mechanisms have been postulated for IVIg and that an anti-inflammatory effect of the sialylated IgG subfraction of IVIg remains to be confirmed in humans. It has for instance been described that IVIg might be contaminated with TGF-ß (84). Nevertheless, further analyses have revealed that TGF-ß contamination cannot explain most of the observed anti-inflammatory functions (85). Furthermore, the galactosylated subfraction of IVIg might also mediate anti-inflammatory functions (35, 50).

### Blocking Abs/Biologica

Other used therapeutic tools are blocking Abs or Biologica that target pro-inflammatory cytokines or their receptors, such as TNFα, IL-6, IL-1, IL-12, IL-23 and IL-17.

Anti-TNFα therapy has probably been developed to reduce local inflammatory immune conditions. However, successful anti-TNFα therapy of RA patients has been shown to increase autoantigen-specific as well as total IgG galactosylation and sialylation levels (86), also assuming an effect on all current (GC) T and B cell responses. The involvement of TNFα in the proper formation of GCs is well known (87). However, the influence of TNFα on certain T_FH_ cell subpopulations and corresponding glycosyltransferases in GC B cells has not yet been verified.

In addition to anti-TNFα application, it was found that treatment of RA patients with tocilizumab, an IL-6 blocking Ab, increased IgG Fc galactosylation levels (88). Recent mouse studies have shown that IL-6 is an important cytokine for inflammatory GC reactions with low B cell intrinsic St6gal1 expression leading to IgG Abs with low galactosylation and sialylation levels (67).

New blocking Abs target IL-12, IL-23 and IL-17, that might inhibit the generation of Th1 and Th17 cells as well as GC T_FH1_ and T_FH17_ cells, which have been shown to be necessary for the induction of IgG Abs with low galactosylation and sialylation levels (67).

Furthermore, whether these new or further cytokine blocking Abs can shift the IgG subclass composition to human IgG4/murine IgG1 to influence inflammatory conditions via this pathway has hardly been examined.

Treatment with corticosteroids also reduce inflammatory conditions and might influence IgG Fc subclass and/or glycosylation shifts.

Currently, the described treatments are applied when inflammatory (auto)immune disease conditions appear. However, in the future, treatments could start earlier when the starting point of the transition from the pre-disease to the inflammatory disease stage is monitored and recognized in IgG autoAb positive “healthy” individuals.

### Nutrition/metabolism

Corticosteroids have unfavorable side effects, and biologic treatment is very expensive. What can autoAb-positive “healthy” individuals do to reduce the probability of undergoing the shift from the autoimmune pre-disease to the inflammatory disease stage (**Figure 1**)? The role of nutrition and metabolism regarding inflammatory conditions has been increasingly discussed lately and could therefore be one possibility to counteract such a shift.

Researchers have found that obesity, a known driver of inflammation (89), increases, whereas extensive weight loss decreases the IgG Fc agalactosylation level (65). Furthermore, a positive correlation between body mass index (BMI) and the IgG Fc agalactosylation level has been recently described in various studies (62, 64). Thus, the metabolic state of an individual seems to influence the inflammatory immune status.

Accordingly, a positive correlation between BMI and the development of several autoimmune diseases has been observed (90–92). Moreover, fasting intervals simultaneously decrease inflammatory disease symptoms and the IgG agalactosylation level in RA patients (93).

More targeted dietary changes also result in improvement of inflammatory (auto)immune diseases (94, 95) and for some inflammatory autoimmune diseases, an influence of diet on T/B cell responses has been described (96–99).

Secondary plant metabolites seem to be able to change an inflammatory state toward more tolerogenic conditions, such as certain phenolic acids that can modulate the production of pro-inflammatory cytokines (100). Moreover, polyunsaturated fatty acids (PUFAs) that occur not only in plants but also in eggs and fish have shown beneficial effects on inflammatory autoimmune diseases such as RA, SLE, multiple sclerosis and diabetes type-1 (101, 102).

Certain diets might also act on the gut bacterial composition and the generation of gut bacterial metabolites. Fasting versus Mediterranean diets change the microbiome composition in RA patients (103). For some of these microbial metabolites, like short chain fatty acids (SCFAs), it is well known that they mediate anti-inflammatory properties and can even influence T/B cell responses. The SCFA butyrate (C4), for instance, reduces IFNγ and inflammatory IL-17 levels (104) and promotes the differentiation of T follicular regulatory cells (105). Furthermore, butyrate induces the generation of IL-10^+^ regulatory B cells (106) and PCs (107) and alters IgG subclass distributions toward less IgG2b (and a tendency toward less IgG2a) in mice (107).

Together, single nutrients and metabolites might have a strong potential to boost the transition to the inflammatory autoimmune disease stage, but others might have the capacity to re-direct inflammatory T/B cell responses or even to hold IgG autoAb-positive “healthy” individuals in the pre-disease stage (**Figure 1**).

### Environment

Another interesting factor that should be considered in the context of inflammatory autoimmune diseases is the environment, such as stress. It is generally believed that stress is a potent inducer of inflammation (108, 109) and, even further, of inflammatory autoimmune diseases (110). In living conditions where stress-levels are generally high, such as shift work, there is growing evidence that the prevalence and disease onset of inflammatory autoimmune diseases is enhanced (111–113). Therefore, it is of interest to determine whether stress can influence the inflammatory status of the T/B cell and the Ab response. Recently, a study with rats investigated the effects of chronic stress on IgG Abs (114). Stress induced higher IgG2a and IgG2b agalactosylation levels in young female rats but higher IgG2b galactosylation levels in older female rats. In the future, addditional research is needed to investigate the influence of stress and other environmental factors on the inflammatory T/B cell and IgG Ab status.

### Conclusion

Several lines of evidence suggest a two-step model for the development of inflammatory autoimmune diseases. In stage one low–/non–inflammatory T/B cell and IgG Ab responses occur that can, but do not have to shift to more inflammatory T/B cell and IgG Ab responses inducing stage two with inflammatory autoimmune disease phenotypes. Early identification and observation of IgG autoAb positive “healthy” individuals might help to recognize changes in the T/B cell and IgG Ab response for starting anti-inflammatory treatments to abolish the transition into stage two. Healthy diets and agreeable environments might help to maintain the less inflammatory stage one. The IgG subclass distributions and IgG Fc glycosylation pattern might thereby act as suitable biomarkers to recognize the transition from stage one to stage two.

## Author contributions

The conceptualization was done by JSB and ME. The review results from the discussion and the consensus of all authors. The review was written by JSB, MB, and ME. All authors contributed to the article and approved the submitted version.

## Funding

This study was supported by the Deutsche Forschungsgemeinschaft [(DFG, German Research Foundation): 429175970 (RTG 2633); 400912066 (EH 221/11-1); and 390884018 (Germany’s Excellence Strategies - EXC 2167, Precision Medicine in Chronic Inflammation (PMI)] (ME). JSB was a PhD student of the RTG 2633.

## Acknowledgments

The graphical element (**Figure 1**) was “Created with BioRender.com” with the agreement of BioRender (#AT246ELXAB).

## Conflict of interest

The authors declare that the research was conducted in the absence of any commercial or financial relationships that could be construed as a potential conflict of interest.

## Publisher’s note

All claims expressed in this article are solely those of the authors and do not necessarily represent those of their affiliated organizations, or those of the publisher, the editors and the reviewers. Any product that may be evaluated in this article, or claim that may be made by its manufacturer, is not guaranteed or endorsed by the publisher.
